# Mechanistic insights and characterization of cardiomyopathy due to Sickle Cell Disease

**DOI:** 10.1186/1532-429X-14-S1-O78

**Published:** 2012-02-01

**Authors:** Amit R Patel, Homaa Ahmad, Ankit A Desai, Thejasvi Thiruvoipati, Kristen M Turner, Lynn Weinert, Chattanong Yodwut, Peter Czobor, Nicole Artz, Sharon Trevino, Victor Mor-Avi, Roberto Machado, Joe G Garcia, Roberto Lang

**Affiliations:** 1 Medicine, University of Chicago, Chicago, IL, USA; 2 Medicine, University of Illinois Chicago, Chicago, IL, USA; 3 Medicine, Mahidol University, Bangkok, Thailand; 4 Medicine, Loyola Medical Center, Maywood, IL, USA

## Summary

We sought to characterize the features of sickle cell cardiomyopathy and to identify causative mechanisms using comprehensive cardiac magnetic resonance, echocardiography, and arterial tonometry. We found that sickle cell cardiomyopathy is characterized by 4-chamber dilation, myocardial fibrosis, abnormal myocardial perfusion reserve, diastolic dysfunction, and only rarely myocardial iron overload. Left ventricular dilation and myocardial fibrosis are associated with increased blood transfusion requirements; where as, diastolic dysfunction is due to increased aortic stiffness.

## Background

Cardiovascular disease is an important cause of morbidity and mortality in adults with sickle cell disease (SCD). We sought to characterize the features of SCD cardiomyopathy and to identify causative mechanisms.

## Methods

Stable adults with SCD (n=38) and matched controls (n=13) were prospectively recruited to undergo 1) multiparametric cardiovascular magnetic resonance (CMR) (i.e. steady state free precession cine, regadenoson first pass myocardial perfusion, phase sensitive inversion recovery late gadolinium enhancement (LGE), and myocardial and hepatic multi-echo time (TE) single breath-hold T2* imaging), 2) echocardiography (TTE), and 3) applanation tonometry (pulse wave analysis). Chamber size and function were measured from CMR using method of disks. Myocardial perfusion reserve index (MPRi) was calculated from time intensity curves generated from first pass perfusion images as the stress to rest ratio of mid-ventricular myocardium upslope (normalized by the left ventricular cavity upslope). LGE was considered present if the signal intensity (SI) was >5 standard deviations above normal remote myocardium and if seen in 2 consecutive slices or 2 imaging planes. Myocardial and hepatic T2* times were calculated using the formula ΔTE/ ln(SI TE2/SI TE1). Presence of diastolic dysfunction (DD) was determined using American Society of Echocardiography criteria. Aortic augmentation index was determined using standard tonometry methods.

## Results

Compared to controls, patients with SCD had severe dilation of the left ventricle (124±27 vs 79±12 ml/m2), right ventricle (127±28 vs 83±14 ml/m2), left atrium (65±16 vs 41±9 ml/m2), and right atrium (78±17 vs 56±17 ml/m2), p<0.001 for all. SCD patients had a 21% lower MPRi than controls (1.47±0.34 vs 1.87±0.37, p=0.03). Twenty-five percent (8/32) of SCD patients (but no controls) had LGE (see figure). One SCD patient had evidence of myocardial iron overload. DD was more commonly present (26% (9/35) vs 8% (1/13)) and filling pressures (E/e’) were higher in SCD patients (9.3±2.7 vs 7.3±2.0, p=0.01). Aortic augmentation index was higher (23.7±17.2 vs 12.6±20.4, p=0.05) in SCD patients. LV dilation and the presence of LGE were inversely correlated to hepatic T2* times (i.e. hepatic iron overload due to frequent blood transfusions), p<0.05 for both; whereas, DD and increased filling pressures correlated with aortic stiffness (augmentation index), p<0.05.

**Figure 1:**
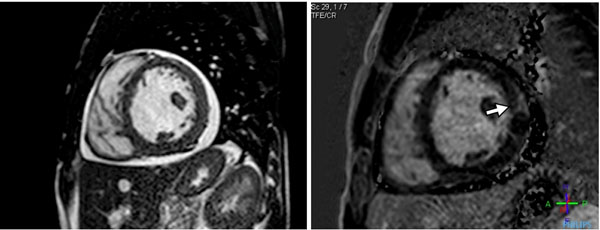


## Conclusions

SCD cardiomyopathy is characterized by 4-chamber dilation, myocardial fibrosis, abnormal MPRi, DD, and rarely myocardial iron overload. Left ventricular dilation and myocardial fibrosis are associated with increased blood transfusion requirements; whereas, DD is due to increased aortic stiffness.

## Funding

Study funded through departmental funds.

